# Reliability of echocardiographic speckle-tracking derived bi-atrial strain assessment under different hemodynamic conditions

**DOI:** 10.1007/s10554-017-1154-7

**Published:** 2017-05-12

**Authors:** Mahdi Sareban, Tabea Perz, Franziska Macholz, Bernhard Reich, Peter Schmidt, Sebastian Fried, Heimo Mairbäurl, Marc M. Berger, Josef Niebauer

**Affiliations:** 1 Institute of Sports Medicine, Prevention and Rehabilitation, Salzburg General Hospital, Paracelsus Medical University, Lindhofstr. 20, 5020 Salzburg, Austria; 2 Department of Anesthesiology, Perioperative and General Critical Care Medicine, Salzburg General Hospital, Paracelsus Medical University, Müllner Hauptstraße 48, 5020 Salzburg, Austria; 3 Department of Anesthesiology, University Hospital Heidelberg, Im Neuenheimer Feld 672, 69120 Heidelberg, Germany; 4 Medical Clinic VII, Sports Medicine; Translational Lung Research Center Heidelberg (TLRC-H), Member of the German Center for Lung Research (DZL), University of Heidelberg, Im Neuenheimer Feld 410, 69120 Heidelberg, Germany

**Keywords:** Cardiac imaging, Deformation, Atrial function, Diastolic function, Reproducibility

## Abstract

The aim of this study was to assess intra- and inter-observer variability of left (LA) and right atrial (RA) strain indices obtained by two-dimensional speckle-tracking echocardiography (2D-STE) in a healthy group of individuals at low-altitude and after rapid ascent to high-altitude in order to provoke altered systemic and pulmonary hemodynamics otherwise seen in various cardiac diseases. Twenty healthy subjects underwent transthoracic echocardiography during a baseline examination at low-altitude (424 m) as well as 7, 20 and 44 h after arrival at high-altitude (4559 m). Atrial strain indices (i.e. reservoir, conduit and contractile strain) were determined off-line by two independent observers. Intra- and inter-observer reproducibility of variables was assessed by intra-class correlation coefficients (ICCs), coefficients of variation and Bland Altman plots. Heart rate, systemic blood pressure and pulmonary artery pressure increased significantly from low-altitude to the first examination at high-altitude. Intra-observer ICCs were ≥0.90 except for RA conduit strain with an ICC of 0.86. The mean intra-observer differences were small and limits of agreement of relative differences were narrow for all atrial strain parameters (<3 and <16%, respectively). Inter-observer ICCs (0.80–0.90), mean biases and limits of agreement (<4 and <20%, respectively) were greater than intra-observer results for all parameters. Intra- and inter-obserer ICCs for all atrial strain variables did not differ between low- and high-altitude. 2D-STE-derived bi-atrial strain indices have excellent intra- and moderate inter-observer reproducibility with no effect of high-altitude-induced hemodynamic changes on reliability results.

## Introduction

Current literature indicates that left (LA) and right (RA) atria are not only passive transport chambers but actively modulate ventricular filling and thereby contribute to global cardiac performance [1]. This task is performed via three repetitive functional phases: during the *reservoir phase* the atria collect venous return, during the subsequent *conduit phase* the atria passively channel blood into the ventricles and during the final *contraction phase* the atria actively pump blood into the ventricles [1, 2]. Transthoracic echocardiography (TTE) depicts the major diagnostic tool for analyzing atrial function [3]. Still, until recently volume and Doppler-derived analyses fell short of sufficiently extracting intrinsic atrial function from ventricular interdependence, let alone measuring the distinct atrial phases. Recent advances in echocardiographic imaging and mainly the advent of two-dimensional speckle tracking echocardiography (2D-STE) myocardial strain assessment enabled a more independent insight into atrial function by visualising and calculating each of the phases separately [4]. Alterations in 2D-STE-derived atrial strain assessment have already been described in patients with systemic arterial hypertension [5, 6], atrial fibrillation [7] and diastolic heart failure [8]. Indeed, this non-invasive tool holds promise for detecting even subclinical stages of atrial dysfunction [9] which would enable initiation of therapy before irreversible atrial damage occurs and thereby has important clinical implications for the evaluation and management of patients with various cardiac diseases. However, before implementing this method into clinical routine its intra- and inter-observer variability has to be assessed. So far, reliability studies had several shortcomings which included incomplete statistics [10, 11], studying intra-observer variability only, not assessing reliability of all phases of atrial function or examining very homogenous cohorts [12]. To this end, we not only performed measurements at low-altitude but used rapid ascent to high-altitude as an intervention in order to provoke heterogeneity in resting physiologic variables like heart rate (HR), systemic blood pressure (BP) and systolic pulmonary artery pressure (PAPs) which are commonly increased in pathologic conditions like systolic and diastolic heart failure. In addition, we performed serial STE-derived atrial strain assessments of all three functional phases, studying both atria and assessing intra- as well as inter-observer variability using relative as well as absolute reliability indices.

## Methods

### Study design and population

Twenty subjects of a high-altitude study (n = 51) assessing the effects of inhaled budesonide on the incidence and severity of AMS after rapid ascent (<24 h) from Alagna (1130 m, Italy) to the Margherita Hut (4559 m, Italy) were randomly assigned to comprehensive 2D echocardiography and assessment for intra- and inter-observer reliability. Prior to the study all participants underwent a comprehensive medical examination at the Institute of Sports Medicine, Prevention and Rehabilitation of the Paracelsus Medical University Salzburg, Austria (424 m) and thereby found to be free from cardiovascular and pulmonary diseases. Echocardiographic examinations were performed during baseline examination as well as 7, 20 and 44 h after arrival at the Margherita Hut. Prior to the study, all subjects gave their written informed consent to participate in the study which was conducted in accordance with the Declaration of Helsinki. The experimental protocol was approved by the ethical review board of the State of Salzburg (Ethics Approval Number: 415-E/1998/13-2016; Clinicaltrials Protocol ID: M2016).

### Echocardiography

All subjects underwent TTE examination using a commercially available ultrasound system (Philips CX50, Phillips Medical Systems, Andover, MA, USA) with a 1.0–5.0 MHz sector array transducer (Philips S5-1, Phillips Medical Systems, Andover, MA, USA). All acquisitions were made by the same experienced echocardiographer with the subject lying in the left lateral decubitus position. Image acquisition for 2D-STE strain analyses was performed from standard transducer positions in accordance with existing recommendations [3]. Image quality was optimized with the focus positioned on the region of interest. Sector depth and width were adjusted in order to maintain a frame rate between 50 and 70 min^−1^. The maximum tricuspid regurgitation velocity was measured in the RV inflow projection of the parasternal long-axis view, the parasternal short-axis view, or the apical four-chamber view and used to calculate the right atrial to right ventricular pressure gradient using the modified Bernoulli equation [13]. For calculation of PAPs, 5 mmHg were added for the estimated RA pressure. HR was measured by electrocardiography connected to the ultrasound system. All images were recorded in a raw Digital Imaging and Communications in Medicine format on a mass storage device.

### Atrial strain analysis

**Fig. 1:**
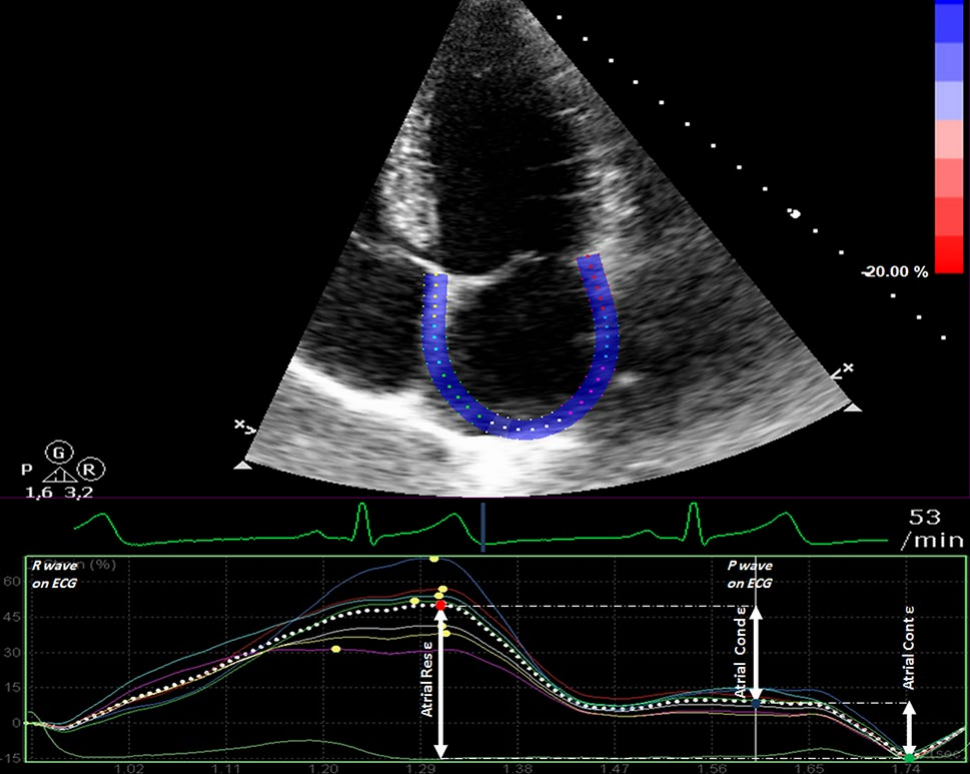
Longitudinal atrial strain (ε) curve using QRS-time analysis in apical 4-chamber view. *Dotted curve* depicts average curve of six segment analysis. The highest point on the curve is the peak atrial ε (*red dot*). This is followed by a plateau and atrial ε at the onset of the P wave on the electrocardiogram (atrial pre-P ε, *blue dot*). The lowest inflection point on the atrial ε curve is the minimal atrial ε (*green dot*). Atrial reservoir ε (Atrial Res ε) is calculated as the difference between peak and minimal atrial ε, atrial conduit ε (Atrial Cond ε) as the difference between peak and atrial pre-P ε and atrial contractile ε (Atrial Cont ε) as difference between atrial pre-P ε and minimal atrial ε, respectively

Images were analyzed offline using a commercially available acoustic tracking software package [QLAB 9 (cardiac motion quantification (CMQ); Phillips Medical Systems]. The region of interest was set at the myocardium using a point-and-click technique and the software divided the atrial wall into six equidistant segments. Orifices of the pulmonary veins, the superior and inferior venae cavae, and segments of inadequate tracing were excluded from further analysis and the remaining segments were averaged. Before processing, a cine loop preview was used to confirm that speckles stayed within the region of interest throughout the cardiac cycle and additional manual adjustment was performed when myocardial tracking was unsatisfactory. If despite manual adjustment speckles of a segment did not stay within the region of interest throughout the cardiac cycle upon cine loop visual control, the segment was excluded from further analysis. If more than two segments had to be excluded, the image results of the individual participant were excluded from the study. The frame at the onset of the R-wave was used as the reference frame. Peak, pre-atrial contraction and minimal strain value were derived from the maximal inflection point, the point correlating with the onset of the P-wave on surface ECG and the minimal inflection point on the LA strain curve. Consequently, 2D-STE-derived atrial reservoir-, conduit- and contractile-strains were calculated as illustrated in Fig. 1. In order to assess intra-observer variability, all 80 TTEs were analyzed twice and 4 weeks apart by the same echocardiographer without any reference to the results of the first analyses. For the assessment of inter-observer variability all TTEs were analyzed by a second observer. Selected cardiac cycles were not marked, so observers had to decide anew which cycles to use.

### Statistical analysis

Intra- and inter-observer reproducibility was assessed by two-way mixed model intra-class correlation coefficient (ICC) [95% confidence interval (CI)] and coefficients of repeatability (CR). The ICC values refer to the thresholds suggested by Vincent et al., indicating <0.8 as poor agreement, 0.80–0.90 as moderate agreement, ≥0.9 as excellent agreement [14]. Although no longer considered appropriate [15], coefficient of variance (CV) was calculated for comparison with other studies. Mean differences and limits of agreement (LoA) were calculated and visualized as Bland–Altman plots for descriptive purposes [16]. Student’s t-test for dependent samples was used to calculate differences between reliability results between low- and high-altitude examinations and statistical significance was assumed for p < 0.05. All statistical analyses were performed using SPSS 21 for Windows (SPSS, Inc., Chicago, IL).

## Results

Baseline characteristics of the study population are shown in Table 1.

**Table 1:** Physical characteristics of the study population

	n = 20
Male	19
Age (years)	33.1 ± 17.8
Body height (cm)	178.2 ± 6.8
Body weight (kg)	73.6 ± 9.2
BMI (kg/m²)	23.1 ± 2.1
HR at rest (bpm)	61.7 ± 11.7
Systolic BP at rest (mmHg)	121 ± 11
Diastolic BP at rest (mmHg)	70 ± 7
LV EF (%)	57.0 ± 6.1
Maximal exercise capacity (W/kg)	4.4 ± 0.5

Values are presented as arithmetic mean ± SD or number of patients (%)

*BMI* body mass index, *HR* heart rate, *BP* blood pressure, *LV EF* left ventricular ejection fraction

**Fig. 2:**
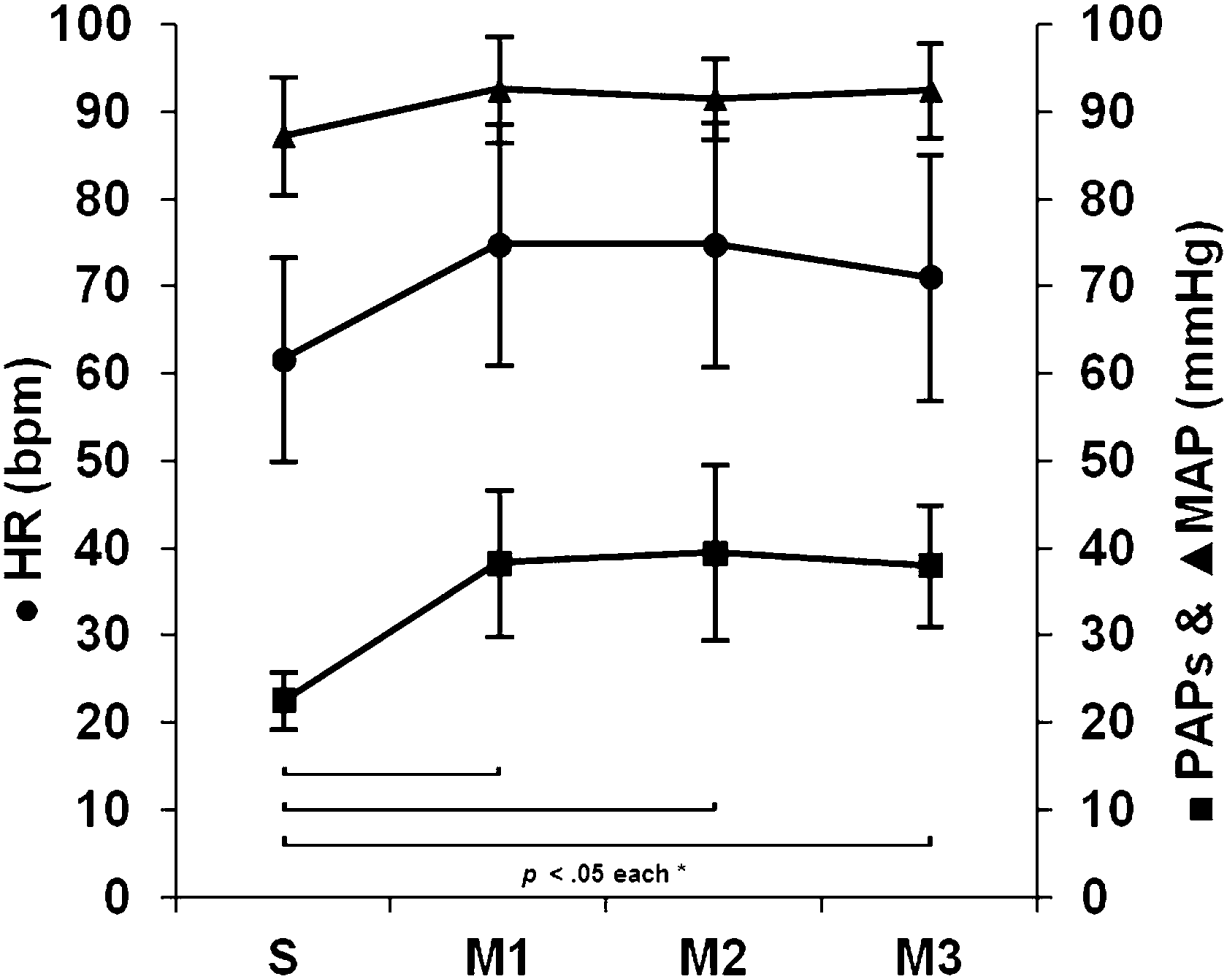
Time course of HR (heart rate), PAPs (systolic pulmonary artery pressure) and MAP (mean arterial blood pressure) from baseline examinations (S), 7 h (M1), 20 h (M2) and 44 h (M4) after arrival at high-altitude, respectively. Mean values ± SD, n = 20. *Significant difference to Salzburg (S); *p* < 0.05

Heart rate, mean arterial blood pressure, and systolic pulmonary artery pressure increased significantly from baseline to the first examination at high-altitude and remained statistically different from baseline examinations during two further echocardiographic studies (Fig. 2).

RA maximal volume increased significantly from low-altitude (53.7 ± 16.6 mL) to the first examination at high-altitude (62.8 ± 21.2 mL, *p* = 0.009) and decreased thereafter without significant difference to baseline at the last examination (57.7 ± 20.9 mL, *p* = 0.151). LA maximal volumes (LAV_low_: 49.0 ± 15.5 vs. LAV_high_: 46.9 ± 13.7 mL; *p* = 0.441) did not change.

Image quality for 2D-STE-derived strain assessment was deemed adequate for further tracking in 96% of images by observer 1 and 99% by observer 2. Frame rate was 58.8 ± 4.2 frames per second which is in line with current recommendations [3].

**Fig. 3:**
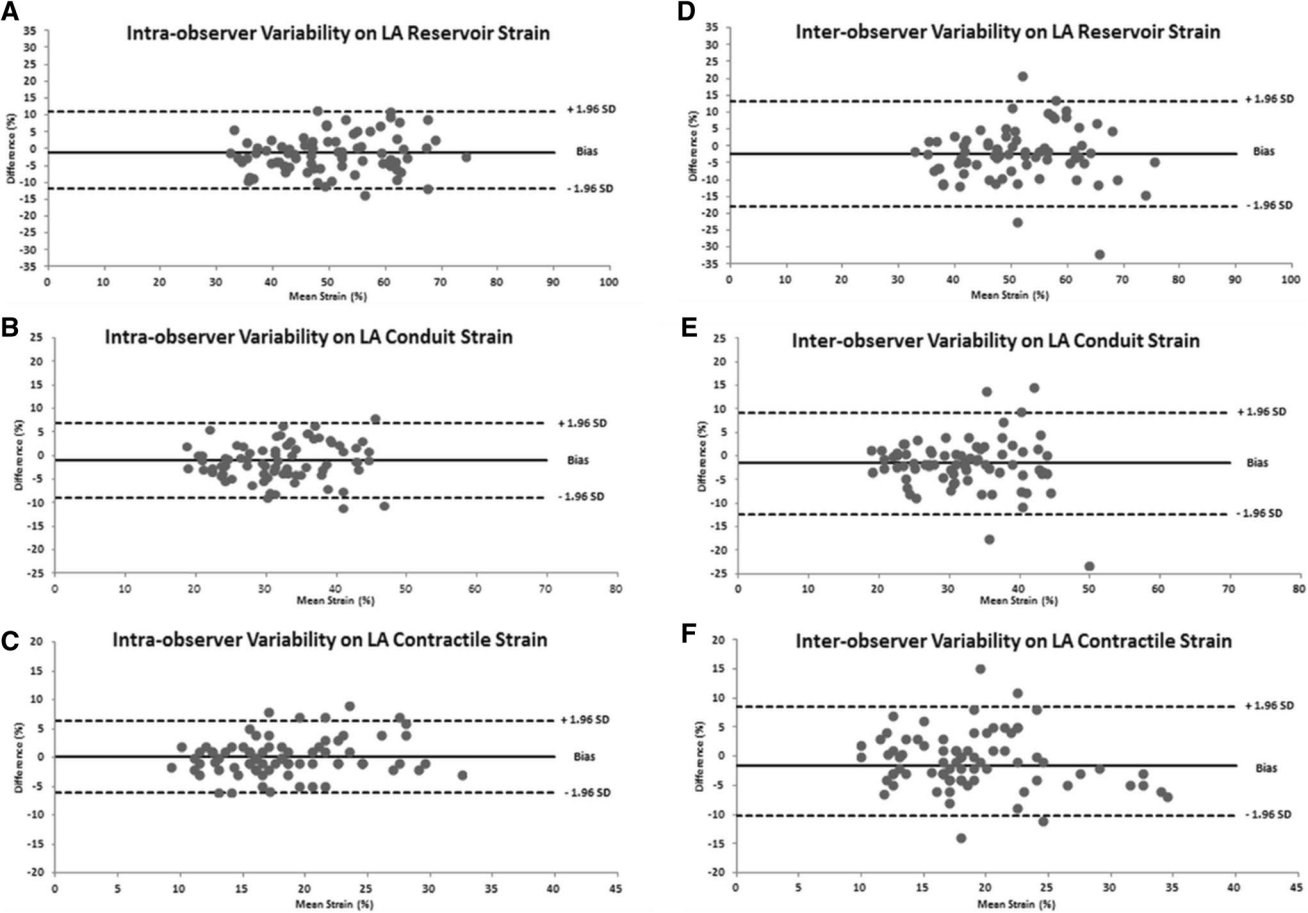
Bland–Altman plots of intra- (*panel*s **a**–**c**) and inter-observer (*panels* **d**–**f**) reproducibiltiy for left atrial (LA) reservior, conduit and contractile strain

**Fig. 4:**
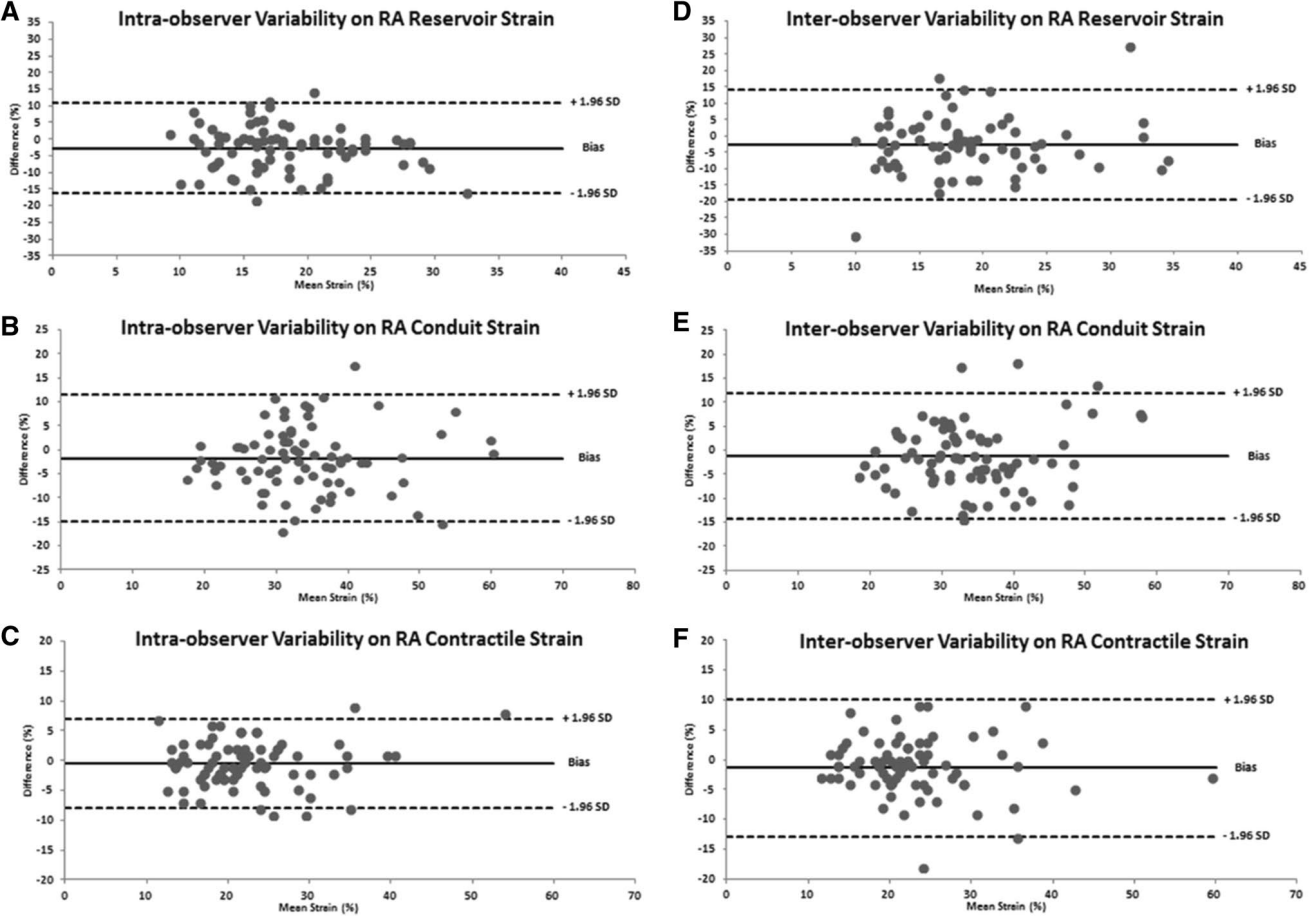
Bland–Altman plots of intra- (*panels* **a**–**c**) and inter-observer (*panels* **d**–**f**) reproducibiltiy for right atrial (RA) reservior, conduit and contractile strain

Mean intra-observer ICCs of all 80 echocardiographic studies were ≥0.90 and CVs < 10% for all analyzed parameters except for RA conduit strain where ICC was 0.86 and CV 11.6% (Table 2). Mean Intra-obserer ICCs for atrial strain variables did not differ when dichotomized in studies obtained from low- and high-altitude (Table 3). Mean intra-observer CRs were <10% for all analyzed parameters except for RA reservoir strain which was 10.6%. The mean intra-observer biases were small and LoAs of relative differences were narrow for all atrial strain parameters (<3 and <16%, respectively; Fig. 3 for LA results and Fig. 4 for RA results).

**Table 2:** Reliability data of atrial strain variables

	Intra-observer					Inter-observer				
	ICC	95% CI	CV(%)	CR(%)	MB(%)	ICC	95% CI	CV(%)	CR(%)	MB(%)
LA Res ε (%)	0.93	0.88–0.95	6.6	±8.7	−1.2	0.86	0.77–0.91	8.5	±12	−2
LA Con ε (%)	0.92	0.88–0.95	7.4	±6.5	−1.1	0.86	0.79–0.91	8.4	±7.6	−1.6
LA Cont ε (%)	0.90	0.85–0.94	9.4	±4.8	0.2	0.82	0.71–0.88	14.6	±7.3	−0.8
RA Res ε (%)	0.92	0.87–0.95	7.1	±11	−2.7	0.86	0.78–0.91	8.5	±13.3	−2.6
RA Cond ε (%)	0.86	0.78–0.91	11.6	±10.6	−1.8	0.85	0.77–0.91	12.4	±11.6	−1.3
RA Cont ε (%)	0.93	0.90–0.96	9.9	±5.8	−0.4	0.87	0.79–0.91	11.7	±7.6	−1.4

*LA* left atrium, *RA* right atrium, *Res* reservoir, *Cond* conduit, *Cont* contractile, *ε* strain, *ICC* intra-class correlation coefficient, *CV* coefficient of variation, *CR* coefficient of reproducibility, *MB* mean bias

**Table 3:** Reliability data of atrial strain variables at low- and high-altitude

	Intra-observer					Inter-observer				
	ICC		CV			ICC		CV		
	Low	High	Low	High	*p* value	Low	High	Low	High	*p* value
LA Res ε (%)	0.91	0.92	6.8	6.5	0.786	0.87	0.85	7.8	8.8	0.599
LA Cond ε (%)	0.94	0.91	7.3	7.5	0.888	0.94	0.83	6.5	9.0	0.542
LA Cont ε (%)	0.87	0.91	9.9	9.4	0.828	0.71	0.84	14.1	14.7	0.845
RA Res ε (%)	0.92	0.91	6.4	7.4	0.567	0.90	0.85	7.2	9.0	0.372
RA Cond ε (%)	0.92	0.84	10.1	12.1	0.326	0.91	0.84	10.0	13.2	0.102
RA Cont ε (%)	0.86	0.94	9.9	9.9	0.993	0.87	0.86	9.7	12.3	0.273

*LA* left atrium, *RA* right atrium, *Res* reservoir, *Cond* conduit, *Cont* contractile, *ε* strain, *ICC* intra-class correlation coefficient, *CV* coefficient of variation, *Low* examinations at low-altitude, *High* examinations at high-altitude

Mean inter-observer ICCs of all 80 echocardiographic studies were between 0.80 and 0.90 and CVs < 15% for all analyzed parameters. Mean inter-obserer ICCs for atrial strain variables did not differ when dichotomized in studies obtained from low- and high-altitude (Table 3). Mean inter-observer CRs were <15% for all analyzed parameters (Table 2). The mean inter-observer differences and LoAs of relative differences were larger than intra-observer results for all atrial strain parameters (<3 and <20%, respectively; Fig. 3 for LA results and Fig. 4 for RA results).

## Discussion

The purpose of the present study was to assess reliability of comprehensive 2D-STE-derived echocardiographic bi-atrial strain measurements, a novel method which holds promise as a non-invasive diagnostic tool for assessing atrial function. The major findigs are threefold: (i) 2D-STE-derived atrial strain assessment by TTE shows excellent intra-observer and moderate inter-observer agreement according to the classification by Vincent et al.; (ii) intra- and inter-observer agreement are higher for LA compared to RA strain parameters; (iii) high-altitude-induced changes in systemic and pulmonary hemodynamic conditions do not compromise intra- nor inter-observer reproducibility.

### Reliability of LA strain

Intra-observer reproducibility of LA strain indices observed in the present study yielded high agreement and was in line with previous studies [12, 17–19]. Oxborough et al. showed almost identical intra-observer ICC and CV of LA reservoir strain of 0.96 and 6%, respectively; inter-observer reliability was not assessed [12]. Cameli et al. depicted an intra-observer CV of 3.6% and inter-observer CV of 4.3% for LA reservoir strain [17]. Still, both trials studied LA reservoir strain only and included a relatively healthy and young cohort with homogenous physiologic variables. In a trial on atrial mechanics in diastolic heart failure with 12 out of 20 randomly selected participants presenting with LA enlargement, the intra-observer CV was 6.3% and ICC 0.86 for LA reservoir strain [18]. In another study on asymptomatic rheumatic mitral stenosis with LA enlargement and increased PAPs intra- and inter-observer CVs for LA reservoir strain were 3 and 5%, respectively [19]. Findings of these seminal studies have rightly set the stage for a wider use of LA strain assessment. Before doing so, however, absolute reproducibility indices as well as Bland–Altman plots are warranted to facilitate clinical interpretation of the reliability data.This hold especially true as recently, LA reservoir strain cut-offs have been suggested to catagorize the severity of ventricular diastolic dysfunction [20]. In the present study in subjects with changing systemic and pulmonary hemodynamic conditions with parallels to patients with ventricular diastolic dysfunction [21], obtained intra- and inter- observer RCs and LoAs raise the question whether the reliability of this method is currently high enough for categorizing diastolic dysfunction by using the proposed cut-off values. Still, regarding the technical limitations of Doppler- and volumetric based functional LA assessment which are the current standard of clinical care, 2D-STE-derived atrial strain method enables a less angle-dependent assessment of intrinsic atrial function. One area of required improvement is inter-overserver reproducibility, which in the present study produced results inferior to intra-oberserver reproducibility with even some outliers. While it is not surprising that there is better agreement with ones own measurements than with those of a different observer, this points to the shortcoming of manual or semi-automated measurements where the human factor leads to higher variability. Also, as observers were free to independtly choose the frames for strain analysis, inter-beat variation was unavoidable, as it would be the case in clinical routine. Lastly, as with all methods, 2D-STE assessment requires a learning curve and since to date this method is predominantly used for research purposes only, it will always be difficult to find a second observer of comparable skills and experience in order to achieve good agreement. A further limitation is the lack of standardization of STE-derived atrial strain assessment. Indeed, standardized atrial strain focused image acquisition, standardized methods for generation of the strain curve as well as standardized training is warranted in order to improve reliability and thereby pave the way for implementing STE-derived atrial function assessment into clinical routine.

### Reliability of RA strain

In general, reliability data of STE-derived RA strain assessment is very scarce but in good agreement with data reported in the present study. Padeletti et al. [6] depicted an intra-observer CV of 9% and inter-observer CV of 8% for RA reservoir strain in ten young and healthy individuals. D’Andrea et al. studying 130 patients with diastolic heart failure, intra-observer Bland–Altmann analyses depicted a mean bias of 3.3% with LoAs of ±1.8 and an inter-observer mean bias of 3.4% with LoAs of ±1.9 for free wall RA reservoir strain, respectively [22]. Of note, the present study yielded larger LoAs than the study conducted by D’Andrea et al. Still, a comparison is difficult since D’Andrea et al. studied the free wall and thereby regional RA reservoir strain only in contrast to the present study which studied global RA strain. Unfortunately, only few controlled atrial strain studies of the RA exist. Sakata et al. showed a reduced RA reservoir strain in patients with pulmonary artery hypertension compared to controls [23]. Concerning these results, our absolute reliability data, intra-observer RCs and LoAs for RA reservoir strain in a cohort including subjects with increased PAPs and enlarged RAs as commonly seen in patients with pulmonary as well as cardiac diseases depict promising reliability for this method to be used for RA function assessment. Still, due to the lack of normal as well as cut-off RA strain values for disease states its clinical use is currently under investigation. For the same reasons as mentioned above for the LA, inter-observer agreement was inferior to intra-observer agreement for all RA phasic strain measurements.

### High-altitude-induced physiologic changes and bi-atrial strain reproducibility

In the present study high-altitude induced mainly physiological changes commonly seen particularly in sub-clinical stages of various cardiac diseases. Especially HR, which was significantly elevated at high-altitude, compromises 2D-STE-tracking quality and requires improved image acquisition and higher frame rates for STE-derived strain analysis [24]. Physiologic alterations at high-altitude did not affect reproducibility in this study and thus underlining the diagnostic potential of this technique in differing physiological conditions.

### Limitations

We used a selected cohort of middle-aged, healthy and fit individuals and thus the current reliability data cannot be generalized to all cardiac patients with potentially inferior echocardiographic window. Still, with ascending to high-altitude we provoked heterogeneity in resting physiologic variables which resemble pathologic conditions in which atrial function assessment plays an important role.

For the generation of the STE-derived atrial strain curve different reference points on the ECG (R-wave or P-wave) can be used based on the software package. The present study used R-wave as a reference point due to software package feasibility in contrast to other studies using a P-wave trigger. Still, atrial phasic values can be measured using both reference points using maximal, minimal and P-wave strain values for calculating phasic atrial strain.

## Conclusion

In this reliability study, 2D-STE-derived bi-atrial strain function indices were found to have an excellent intra- and moderate inter-observer reproducibility with superior intra- and inter-observer agreement for LA compared to RA strain parameters. High-altitude-induced changes in hemodynamic parameters did not compromise intra- nor inter-observer reproducibility. Overall, application of 2D-STE appears to be a reliable method to study atrial function. Thus, its measurement may further enhance our understanding of atrial mechanics and possibly improve clinical care.
